# Transverse mesocolic hernia successfully treated by emergent laparoscopic reduction in a geriatric patient: A case report

**DOI:** 10.1016/j.ijscr.2025.111902

**Published:** 2025-09-04

**Authors:** Noriya Takayama, Osamu Takata, Daisuke Ishioka, Ichiro Imai, Yoko Yoshida, Sakae Sekiya

**Affiliations:** Arai Hospital, 2-2-28, Kukichuo, Kuki-shi, Saitama, 346-0003, Japan

**Keywords:** Internal hernia, Transverse mesocolic hernia, Emergent surgery, Laparoscopic reduction, Geriatric, Case report

## Abstract

**Introduction and importance:**

Transverse mesocolic hernia is an extremely rare type of internal hernia, with only a limited number of cases reported to date. In this case report, we present a geriatric patient with a transverse mesocolic hernia who was successfully treated with emergent laparoscopic surgery.

**Case presentation:**

An 89-year-old male patient presented with abdominal pain and distension that had begun the previous day. He had multiple comorbidities, such as diabetes mellitus, hypertension, and prostate cancer. Additionally, he had been prescribed an antiplatelet agent for a history of cerebral infarction. He had no previous abdominal surgery except for an appendectomy at a young age. Computed tomography revealed a closed-loop obstruction of the ileum on the right side of the superior mesenteric vein. Emergent laparoscopy revealed incarceration of the small intestine through a defect in the transverse mesocolon, which was successfully reduced. The postoperative course was uneventful, and the patient was discharged without any decline in activities of daily living.

**Clinical discussion:**

First, this was an extremely rare case of internal hernia that required emergent surgical intervention. Second, minimally invasive surgery was successfully performed, contributing to a rapid postoperative recovery, despite the patient being geriatric and having multiple surgical risk factors.

**Conclusion:**

We managed a case of transverse mesocolic hernia. This case report provides valuable insights into the clinical course of the disease and the effectiveness of emergent laparoscopic reduction in geriatric patients.

## Highlights

• An 89-year-old male patient was diagnosed with a bowel obstruction.
• Computed tomography revealed a closed-loop obstruction of the ileum.
• Laparoscopy confirmed a transverse mesocolic hernia, which was successfully reduced.
• The patient was discharged home without any decline in activities of daily living.
• This case offers insights into treating a very rare internal hernia in geriatric patients.

## 1 Introduction

Transverse mesocolic hernia is an extremely rare type of internal hernia. Its true incidence is difficult to determine, as only a limited number of cases have been reported to date. The etiology is considered to include congenital abnormalities, trauma, circulation disorders, and postoperative causes [1,2]. Due to its rarity, most cases are not diagnosed preoperatively; however, they typically require emergent surgical intervention for hernia reduction. In this case report, we present a geriatric patient with a transverse mesocolic hernia who was promptly treated by emergent laparoscopic reduction without any decline in activities of daily living (ADL).

## 2 Methods

The work has been reported in line with the SCARE criteria [3].

## 3 Presentation of case

**Fig. 1:**
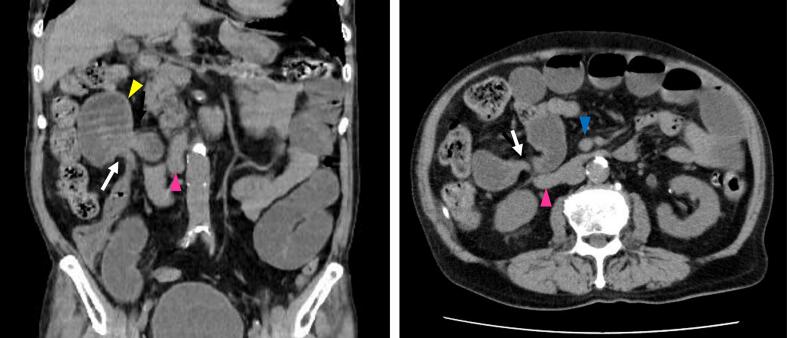
The CT scan demonstrates dilatation of the small bowel and identifies the site of intestinal obstruction (white arrow) with closed-loop (yellow arrowhead) located on the right side of the superior mesenteric vein (blue arrowhead) and anterior to the third portion of the duodenum (pink arrowhead). (For interpretation of the references to colour in this figure legend, the reader is referred to the web version of this article.)

**Fig. 2:**
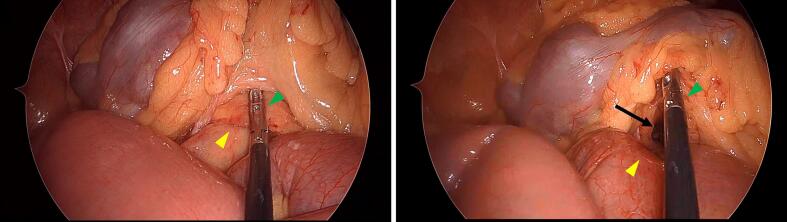
Left image: The incarcerated intestinal mesentery (yellow arrowhead) and the edge of the transverse mesocolon defect grasped with laparoscopic forceps (green arrowhead). Right image: The incarcerated intestine (yellow arrowhead) after successful reduction. The defect in the transverse mesocolon is clearly visible (black arrow). (For interpretation of the references to colour in this figure legend, the reader is referred to the web version of this article.)

**Fig. 3:**
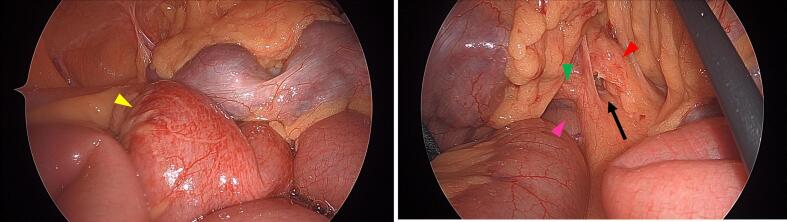
Left image: The incarcerated intestine (yellow arrowhead), approximately 10 cm in length, exhibited erythema due to venous congestion. Right image: The defect in the transverse mesocolon (black arrow), located to the right of the ligament of Treitz and anterior to the third portion of the duodenum (pink arrowhead), is identified between the right colic artery (green arrowhead) and the middle colic artery (red arrowhead). (For interpretation of the references to colour in this figure legend, the reader is referred to the web version of this article.)

An 89-year-old male patient presented as a walk-in with abdominal pain and distension that had begun the previous day. He had multiple underlying conditions, including diabetes mellitus, hypertension, and prostate cancer managed with hormone therapy. Additionally, he had been prescribed an antiplatelet agent (clopidogrel) for a history of cerebral infarction and prostaglandin E1 (limaprost alfadex) for lumbar spinal canal stenosis. However, he had no sequelae of cerebral infarction and his ADLs were fully maintained. He had no history of prior abdominal surgery except for an appendectomy at a young age. Vital signs were stable, and he was afebrile. Physical examination revealed abdominal distension and mild tenderness, without any muscular guarding or rebound tenderness. Laboratory tests showed no significant abnormalities, including no evidence of metabolic acidosis or intestinal ischemia, except for slight hypoxia with mild respiratory alkalosis (pH 7.489, pO_2_ 77.3 mmHg, pCO_2_ 33.8 mmHg, HCO_3_^−^ 25.1 mmol/L) and signs of dehydration (BUN 24 mg/dL). Computed tomography (CT) demonstrated small bowel dilatation with a closed-loop obstruction (Fig. 1). The site of obstruction was located on the right side of the superior mesenteric vein (SMV), adjacent to the ventral aspect of the third portion of the duodenum. The patient was diagnosed with small bowel obstruction due to internal hernia incarceration. As the patient was geriatric and presented with multiple surgical risks, there was a potential for postoperative decline in ADLs. However, spontaneous resolution through conservative management was deemed unlikely. Laparoscopic surgery was considered favorable, as there was no evidence of necrosis based on vital signs, physical examination, and laboratory findings. Therefore, emergent laparoscopic intervention was undertaken. Intraoperative findings revealed incarceration of the small intestine through a defect in the transverse mesocolon. The incarcerated intestine was successfully reduced using laparoscopic forceps without the need for enlarging the hernia orifice (Fig. 2). Approximately 10 cm of the intestine had been incarcerated. The segment appeared edematous but showed no signs of ischemia, and bowel resection was not required. The defect in the transverse mesocolon was located to the right of the ligament of Treitz and identified between the right colic artery (RCA) and the middle colic artery (MCA) (Fig. 3), confirming the diagnosis of a transverse mesocolic hernia. The hernia orifice was not closed in this case, as there was an inability to safely proceed with orifice closure for the following reasons. First, marked intestinal dilatation and extensive adhesions on the right abdominal wall were present, and attempting defect closure was anticipated to considerably prolong the operative time. Second, tissue fragility due to advanced age, combined with the use of blood thinners, would have increased the risk of bleeding with more extensive tissue manipulation. Therefore, a minimally invasive and expedited procedure avoiding laparotomy was deemed more appropriate for this geriatric patient with multiple comorbidities. The operative time was 36 min, with minimal blood loss (<5 mL). The postoperative course was uneventful. Oral intake was resumed on postoperative day four, and the patient was discharged without any decline in ADLs. No complications or recurrent bowel obstruction were observed during the two-year follow-up period after discharge.

## 4 Discussion

We managed a case of transverse mesocolic hernia. This case report presents the clinical course of a rare internal hernia and provides valuable insights into the effectiveness of emergent laparoscopic reduction in a geriatric patient with multiple comorbidities.

First, transverse mesocolic hernia is an extremely rare form of internal hernia [1,2,4] and is often difficult to diagnose preoperatively [2]. Nevertheless, most cases require emergent surgical intervention to prevent necrosis of the incarcerated intestine. A history of prior abdominal surgery, especially gastric surgery with Roux-en-Y reconstruction [5] or transverse colectomy [6], or the presence of intestinal malrotation, is considered an important predisposing factor for postoperative or congenital mesenteric defects [1,2]; however, this patient had neither a surgical history nor evidence of intestinal malrotation. In this case, CT imaging revealed a closed-loop obstruction of the intestine, prompting the decision to proceed with emergent surgery, although a definitive preoperative diagnosis could not be established. A left paraduodenal hernia, with its orifice located at the fossa of Landzert, to the left of the ligament of Treitz and posterior to the inferior mesenteric vein, was considered in the differential diagnosis. However, intraoperatively, the hernia defect in the transverse mesocolon was located to the right of the ligament of Treitz and identified between the MCA and the RCA. Based on its anatomical location, this case was diagnosed as a transverse mesocolic hernia.

Second, emergent laparoscopic reduction was promptly performed for an 89-year-old geriatric patient with multiple comorbidities, resulting in a rapid recovery without any decline in ADLs. The patient had significant surgical risk factors, including diabetes mellitus, hypertension, a history of cerebral infarction, and the use of antiplatelet agents and prostaglandin E1. However, based on CT findings suggestive of internal hernia, emergent surgical intervention was deemed necessary. Given the absence of peritoneal irritation on physical examination and the lack of evidence of intestinal ischemia on laboratory evaluation, laparoscopic surgery was considered appropriate. As a result, the hernia orifice was identified and successfully reduced. In this case, closure of the hernia orifice was not performed, although orifice closure is typically undertaken in most laparoscopic cases [7,8]. This decision was based on the patient's risk factors. Marked dilatation of the proximal small intestine and extensive adhesions on the right abdominal wall were present, which would have necessitated adhesiolysis to achieve an adequate surgical view for suturing and additional port placement, thereby considerably prolonging the operative time. Furthermore, tissue fragility due to advanced age, combined with the use of blood thinners, could have increased the risk of bleeding and necessitated a laparotomy. If these conditions had not been present, closure of the hernia orifice would have been performed. However, given the patient's surgical risks in this case, a minimally invasive and expeditious procedure without suture closure was considered more appropriate. At the time of admission, the patient was fully independent with a perfect Barthel index score which is widely used to evaluate a patient's ability to perform basic ADLs [9]. Furthermore, preoperative assessment of physical function has been reported to predict postoperative complications in elderly patients undergoing abdominal surgery [10]. In this case, although the patient was geriatric and had multiple comorbidities, his ADL status was fully independent. As a result, he was successfully treated without any decline in functional capacity.

The clinical course of this case provides valuable insights for surgeons into the management of an extremely rare transverse mesocolic hernia and one of the optimal treatment approaches.

## 5 Conclusion

We managed a case of transverse mesocolic hernia. This provides valuable insights into the clinical course of the disease and the effectiveness of emergent laparoscopic reduction in geriatric patients.

## Author contribution

Study conception and design; Takayama.

Data acquisition and interpretation; Takayama.

Surgery and patient follow-up; Takayama, Takata and Sekiya.

Preparation of images; Takayama.

Drafting the paper; Takayama.

Critical revision; Takayama, Takata, Ishioka, Imai, Yoshida, Sekiya.

Final approval of submitting the manuscript; Takayama, Takata, Ishioka, Imai, Yoshida, Sekiya.

## Consent

Written informed consent for the publication of this case report and accompanying images was obtained from the patient. The signed consent form can be provided to the editors of this journal on request.

## Ethical approval

This study was deemed exempt from ethical approval at our institution, as this paper reports a single case encountered during normal surgical case and does not represent a First-in-Man case report.

## Guarantor

Noriya Takayama, the corresponding author of this paper.

## Research registration number

Not applicable. No research study involved.

## Funding

This research did not receive any specific grant from any funding agency in the public, commercial, or not-for-profit sectors.

## Conflict of interest statement

All authors declare that there is no conflict of interest.
